# Seasonal Movements, Aggregations and Diving Behavior of Atlantic Bluefin Tuna (*Thunnus thynnus*) Revealed with Archival Tags

**DOI:** 10.1371/journal.pone.0006151

**Published:** 2009-07-07

**Authors:** Andreas Walli, Steven L. H. Teo, Andre Boustany, Charles J. Farwell, Tom Williams, Heidi Dewar, Eric Prince, Barbara A. Block

**Affiliations:** 1 Tuna Research and Conservation Center, Stanford University, Hopkins Marine Station, Pacific Grove, California, United States of America; 2 Department of Wildlife, Fish and Conservation Biology, University of California Davis, Davis, California, United States of America; 3 Nicholas School of the Environment and Earth Sciences, Duke University, Durham, North Carolina, United States of America; 4 Monterey Bay Aquarium, Monterey, California, United States of America; 5 National Marine Fisheries Service, Southwest Fisheries Science Center, La Jolla, California, United States of America; 6 National Marine Fisheries Service, Southeast Fisheries Science Center, Miami, Florida, United States of America; University of Aberdeen, United Kingdom

## Abstract

Electronic tags were used to examine the seasonal movements, aggregations and diving behaviors of Atlantic bluefin tuna (*Thunnus thynnus*) to better understand their migration ecology and oceanic habitat utilization. Implantable archival tags (n = 561) were deployed in bluefin tuna from 1996 to 2005 and 106 tags were recovered. Movement paths of the fish were reconstructed using light level and sea-surface-temperature-based geolocation estimates. To quantify habitat utilization we employed a weighted kernel estimation technique that removed the biases of deployment location and track length. Throughout the North Atlantic, high residence times (167±33 days) were identified in four spatially confined regions on a seasonal scale. Within each region, bluefin tuna experienced distinct temperature regimes and displayed different diving behaviors. The mean diving depths within the high-use areas were significantly shallower and the dive frequency and the variance in internal temperature significantly higher than during transit movements between the high-use areas. Residence time in the more northern latitude high-use areas was significantly correlated with levels of primary productivity. The regions of aggregation are associated with areas of abundant prey and potentially represent critical foraging habitats that have seasonally abundant prey. Throughout the North Atlantic mean diving depth was significantly correlated with the depth of the thermocline, and dive behavior changed in relation to the stratification of the water column. In this study, with numerous multi-year tracks, there appear to be repeatable patterns of clear aggregation areas that potentially are changing with environmental conditions. The high concentrations of bluefin tuna in predictable locations indicate that Atlantic bluefin tuna are vulnerable to concentrated fishing efforts in the regions of foraging aggregations.

## Introduction

Atlantic bluefin tuna are large, highly migratory, endothermic fish [1]. They occur throughout the North Atlantic, including the Gulf of Mexico and the Mediterranean Sea and can migrate as adults into sub polar seas. Atlantic bluefin tuna fisheries' catches have reached historic highs in the past two decades, and overfishing has reduced western Atlantic population sizes of mature bluefin tuna by 90% since 1970 [2]–[3].

Recently, electronic tagging studies have provided information on the movements of bluefin tuna in the western and eastern Atlantic [4]–[9]. These studies have demonstrated linkage of western tagged fish between the waters offshore of North Carolina, the Northwest Atlantic and Mediterranean Sea [7], [10], and the Gulf of Mexico during spawning season [9]–[11]. Many studies have used fisheries-independent pop-up satellite archival tag (PSAT) technology, which provide tracks of 1–9 month duration. Problems of premature tag shedding shortens tracking duration and biases positions to the western Atlantic [10], [12]. Another complexity in the interpretation of the PSAT results is that the tagging studies have been conducted on different year classes at various tagging locations.

On the other hand, archival tags provide the capacity to track fish over multiple years [5], [10], which can reveal subtle changes and ontogenety in movement patterns. Based on longitude data and recapture positions from implantable electronic archival tags, Block et al. [5] was able to describe four movement patterns of western tagged bluefin tuna. They were shown to reside in the western Atlantic for one to three years post-release before moving into spawning grounds in the Gulf of Mexico, Bahamas/Carribean or Mediterranean Sea. Some western tagged fish remained outside the known spawning grounds [4], [5], [7], [10,]. Using longitude and latitude estimates derived from both archival and PAT tag data, Block et al. [10] demonstrated the difference in spatial coverage between PSATs and archival tags, with archival tags being able to deliver multiple year tracks which revealed ontogenetic changes in migration patterns.

Many aspects of the ocean-scale migratory biology and behaviors of bluefin tuna remain unknown, in particular the extent and location of foraging grounds and quantification of residence times throughout the Atlantic Ocean. From stomach content studies, Atlantic bluefin tuna are known to be opportunistic feeders [13]–[16] with many species of fish, squid, and crustaceans in their diet. However, as highly mobile and migratory pelagic predators, Atlantic bluefin tuna are likely to optimize their movements to improve their foraging efficiency across regional and ocean basin scales to adapt to the spatiotemporal variability in prey abundance. To satisfy their high energetic demands, bluefin tuna are hypothesized to make long migrations to take advantage of the most productive regions in the oceans [5], [16], [17], [18].

In this study, we examine oceanic movements of archival-tagged bluefin tuna (n = 106) tracked with geolocation estimates [19] between 1996–2006. We analyze patterns of spatial distribution of western tagged individuals throughout the Atlantic with respect to migration movements, season, year, age and origin. To remove biases of deployment location and various track length, the position dataset is weighted by the tracking effort for each unit area. The specific objective is to determine the extent, duration and composition of seasonal aggregations. Moreover, we examine whether bluefin tuna in high-use areas exhibit site-specific diving behaviors and experience site-specific internal/ambient temperatures, and whether their presence coincides with unique biophysical settings that would indicate their importance as foraging habitats. We therefore analyze water temperatures and diving behavior within the high-use areas as well as outside as measured through electronic tags. We explore the monthly conditions of sea surface temperature and derived primary productivity estimates in relation to the presence of tracked bluefin tuna within the high-use areas. In addition, we examine the overall diving behavior in relation to the structure of the water column. The importance of our findings is briefly discussed with regard to migration and foraging ecology and their implications for fisheries management.

## Materials and Methods

### Ethics Statement

The research presented in this manuscript was conducted according to protocols approved by the Stanford University Administrative Panel on Laboratory Animal Care.

### Archival Tagging

**Figure 1:**
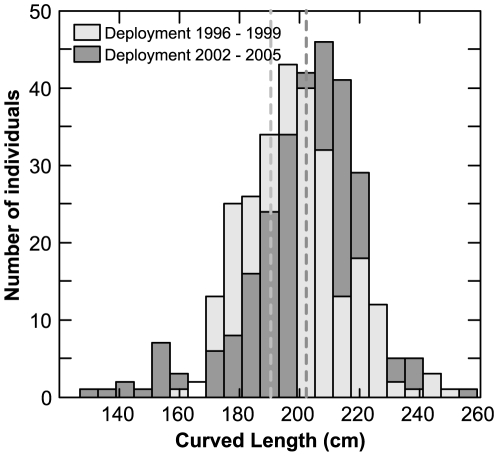
Size distribution of tagged bluefin tuna at deployment between 1996–1999 (mean±sd; 198.3±16 cm CFL; n = 280; light grey bars), and 2002–2005 (203.2±19 cm CFL; n = 281; dark grey bars). Dotted lines indicate corresponding means.

Archival tags (n = 561) were deployed in bluefin tunas tagged and released offshore of Morehead City, North Carolina, USA, between January to March from 1996 to 2005 (approx. 34.5°N and 76.3°W, Figure 1, Table 1) according to the methods previously reported [8], [10], [20].

**Table 1:** Deployment, recovery and position summary.

	1996	1997	1998	1999	2000	2001	2002	2003	2004	2005	2006	Total
**Individuals released**	10	160	x	110	x	x	18	104	94	65	x	**561**
**Individuals recovered by deployment year**	2 (20%)	46 (29%)	x	33 (30%)	x	x	2 (11%)	16 (15%)	6 (6%)	1 (2%)	x	**106**
**Individuals recovered by recovery year**	x	3 (2%)*	8 (6%)*	13 (9%)*	21 (16%)*	11 (20%)*	7 (22%)*	12 (19%)*	13 (18%)*	14 (18%)*	3 (19%)*	**106**
**Longitude days** ** **in Western residency**	290 (n = 2)	1,083 (n = 47)	481 (n = 12)	2,634 (n = 44)	403 (n = 27)	147 (n = 14)	246 (n = 10)	2,343 (n = 27)	940 (n = 21)	318 (n = 17)	3 (n = 3)	**8,885**
**Longitude** ** **days trans-Atlantic**	2 (n = 1)	2 (n = 1)	8 (n = 4)	725 (n = 7)	572 (n = 6)	388 (n = 3)	339 (n = 6)	420 (n = 8)	508 (n = 7)	74 (n = 2)	x	**3,038**

* Cumulative recovery rate to date.

** Lightlevel Geolocations, deployment & recovery positions.

In brief, the tuna were caught using rod and reel techniques. They were brought into the vessel and onto a wet vinyl mat, irrigated with a deck hose with flowing seawater. During surgery, eyes of the fish were covered, and an incision made in the peritoneal cavity with a #22 stainless steel blade. An archival tag (model information below) was implanted into the tuna. Two green and white conventional Floy tags (Floy Tags Inc.) were inserted into the base of the second dorsal fin on both the right and left side. The conventional tags had contact information that alerted the fishers to the presence of the internal electronic tag. A GPS position was recorded from a receiver on the boat, at capture and release of the fish.

#### Archival Tags

Three tag models were used in deployments: Mk7 (Wildlife Computers; 1996–1999), NMT (Northwest Marine Technology; 1996–2002) and LTD2310 (Lotek; 2002–2005). The NMT and the LTD2310 had a stainless steel loop secured to the stainless steel tag case. The loop was used to anchor the tag to the inner surface of the peritoneal cavity, using either a non-dissolvable suture (Ethilon: 4.0 metric nylon suture, with CPX needles size ½, 45 mm diameter or black monofilament, 30″ (75 cm) taper CTX ). We also used a “button technique” in which a modified Floy tag was tied to the stainless steel loop and attached externally with a nylon head into the ventral muscle from the outside of the fish (n = 226).

The NMT tags were set to record the ambient and internal temperatures, pressure and light levels every 128 s during the initial two months. In addition, for the duration of up to 5 years, this brand of tags binned the time series data into temperature and depth histograms, recording the time at depth (1 m bins at 0 to 255 m and 3 m bins at 256 to 765 m) and time at temperature (0.2°C bins from −1.0 to 34°C).

The Mk7 tags were set to log the ambient and internal temperatures, pressure and light levels every 120 sec. providing a maximum record of up to 2 years. Depth is recorded with a resolution ranging from ±1 m (0 to 99.5 m) to ±16 m (500 to 1000 m), and the ambient temperatures with a resolution of 0.1°C in the range from 12.00 to 26.95°C and 0.2°C from 3.00 to 11.95°C, and 27.00 to 37.95°C.

The newer Lotek LTD2310 was set to log every 120 sec., which can potentially yield up to 3960 days of time series data. Depth is recorded with a resolution of 1 m (0–2000 m) and the ambient and internal temperatures with a resolution of 0.05°C (0 to 30°C).

### Geolocation

#### Longitude estimates

The estimation of daily longitudes from the recorded light level data for the three tag models used is described in [10] and Teo *et al.* [20]. Longitude estimates that showed movements of more than three degrees per day were considered biologically unrealistic and were removed using a modified version of the iterative forward/backward-averaging filter [23].

#### Latitude estimates

We used an SST-based method to obtain an improved estimate of daily latitudes as described in detail by Teo *et al.* [8], [20]. The remotely sensed SST data were weekly-averaged MODIS and AVHRR Pathfinder datasets (ftp://podaac.jpl.nasa.gov) at 4 and 9 km respectively. If cloud cover in the search area was greater than 70% for a given day, interpolated MCSST satellite imagery (9 km) was used to estimate latitude.

Estimation of SST-based geolocations was limited to recovered tags that had a continuous record of light level, depth and ambient temperature (Table S1).

The light- and SST-based geolocation methods are affected by decay of sensors, the influence of the diving behavior on light curves, availability of SST's, cloud cover in the remotely sensed SST fields, as well as removal of positions during quality checking [10], [19], [26], [27]. The pressure data in some of the archival tags (Mk7 and LTD2310) drifted from initial calibrations and had to be compensated for sensor drift prior to further analysis. The bluefin tunas were assumed to have reached the surface (<2 m) at least once a day and a third-order polynomial was fitted to the minimum depth of each day of the track. The polynomial was then used to correct the pressure data of the tag by subtracting the polynomial from the raw pressure data. Since the NMT tags only recorded time series data for the initial two months, we were unable to detect or compensate for any long-term drift in the pressure sensors. A decay of the light sensors was not detected. The diving behavior can influence the light curves to such a degree that the estimation of the longitude becomes either unreliable or is not possible. The prior was dealt with the longitude filter described previously. These known problems limit the spatial resolution and accuracy of the positional dataset obtained [19], [20]. To maximize the spatial information obtained, while minimizing the influence of erroneous geolocations, we peformed the spatial analysis steps described in the following section (steps 1–6).

### Analyses

#### Spatial distribution

**Figure 2:**
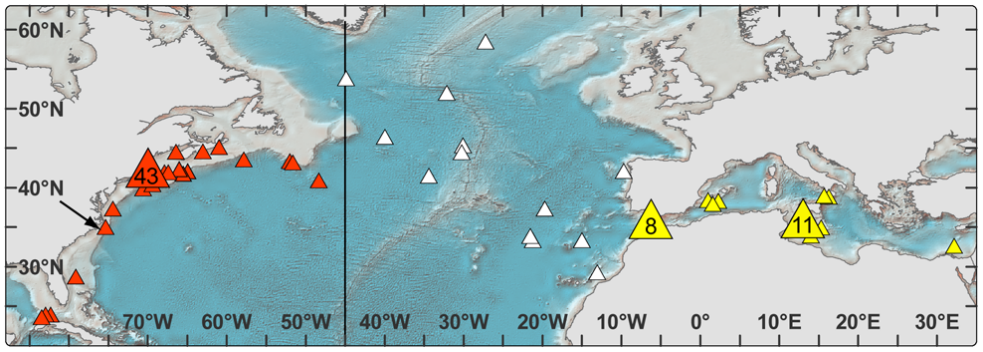
Recovery positions of electronic archival tags (triangles) in western Atlantic (orange, n = 64, 226±21 cm CFL), eastern Atlantic (white, n = 13, 218.4±13 cm CFL), and Mediterranean Sea (yellow, n = 29, 234±17 cm CFL). Line at the 45°meridian indicates the management line. Black arrow indicates location of tag deployments in North Carolina. Large triangles with inset indicate locations of multiple recoveries. The highest recapture rates for these western tagged bluefin tuna were obtained from the region off New England (48% of total recaptures) followed by the Mediterranean Sea (27%). The Central and Northeast Atlantic emerged as the third area of high recovery (13%).

For each geolocation and recovery position (Table 1, Figure 2) of the tracked bluefin tuna, corresponding size of the fish for the date (based on the release length and time at liberty) was estimated based on putative natal origin as identified by the criteria in Block *et al.* [10] and by genetic techniques [23]. The age-length relationships determined by Turner & Restrepo [29] for western Atlantic bluefin tuna and by Cort [30] for eastern Atlantic bluefin tuna were used to calculate their respective length.

Movement patterns were classified into western resident (<45°W) or transatlantic (>45°W) based on the annual migration of an individual as determined through longitude data. To assess the variation in longitudinal distribution between years within each movement pattern, we calculated a coefficient of variation (CV). We first calculated the CV for all longitudes between years and used the mean CV for a given movement pattern along with the number of samples to obtain a corrected CV* [31].

Kernel density estimators have been successfully used in several tracking studies to describe habitat use and identify high use areas for marine animals [10], [32]–[34]. However, when using this technique to quantify utilization distributions from tracking data care needs to be taken to consider biases, ensure transparency and objectivity.

**Figure 3:**
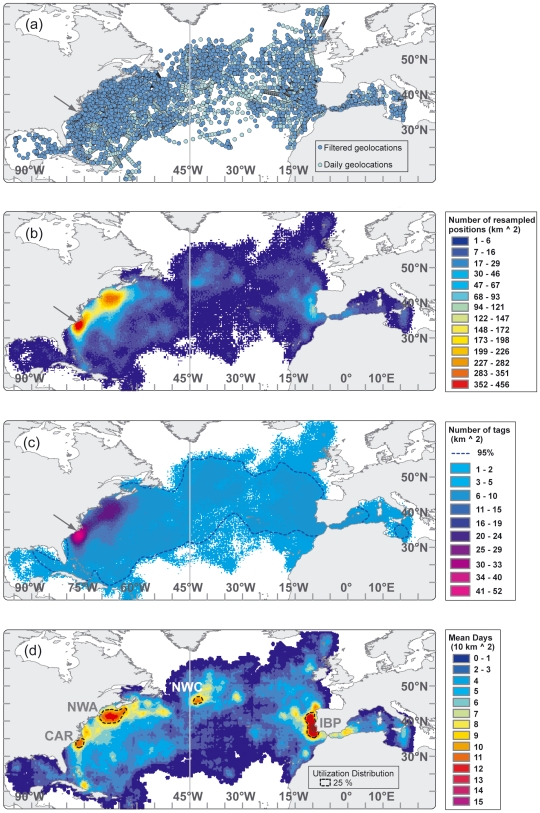
Maps showing calculation of utilization distribution from pooled geolocation tracks. Dark grey line at 45° meridian indicates management line. (a) Blue circles are all deployment, daily geolocation and recapture positions (n = 7,793) from 106 bluefin tuna between 1996–2006 and light blue circles indicate daily, linearly interpolated positions (n = 14,716) (b) Kernel density grid of resampled daily positions (n = 1,471,600). (c) Grid of number of bluefin tuna tracked per square kilometer. Blue line outlines area of ≥3 tags. (d) Normalized kernel density grid of number of daily geolocations weighted by number of fish tracked per unit area. Black, dotted line outlines 25% utilization distributions, showing four regions of high residency throughout the North Atlantic.

**Figure 4:**
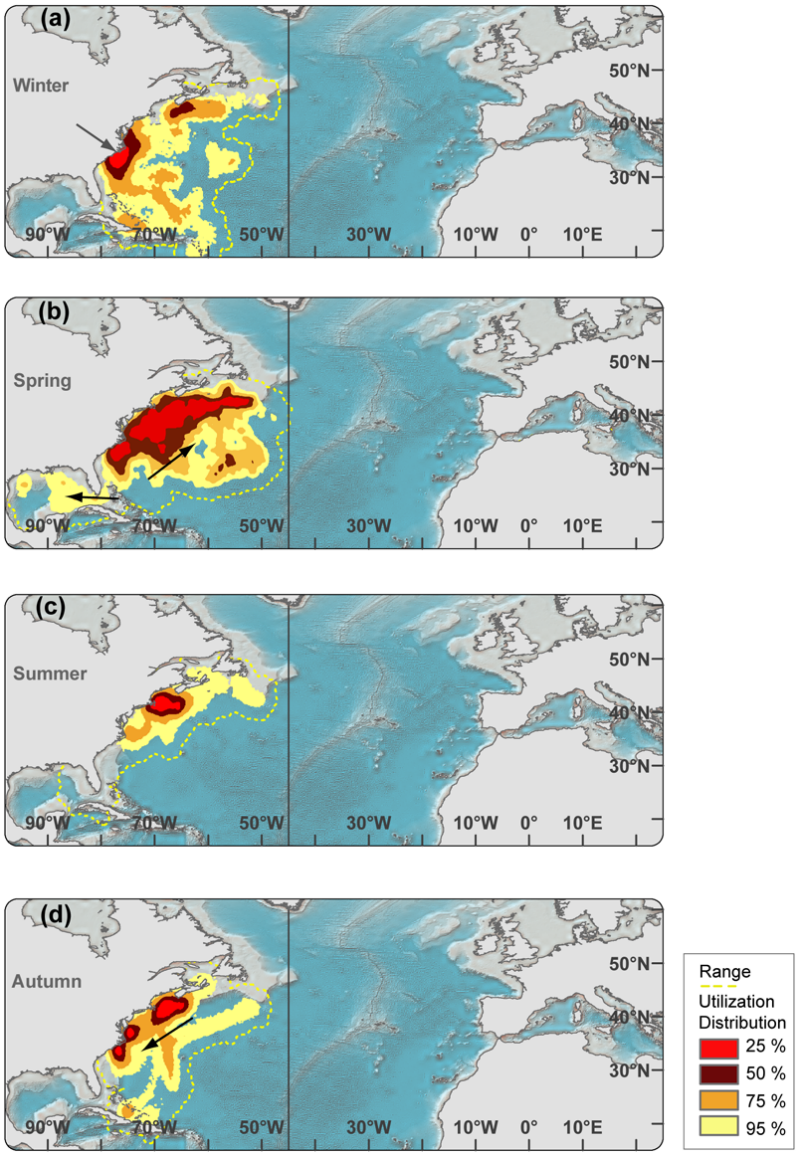
Seasonal utilization distributions of bluefin tuna in *western resident* migration cycle (n = 49, 224±16 cm CFL). Black arrows in ocean depict general direction of movements during relevant season. a) Winter. Grey arrow in North Carolina depicts approximate deployment location. b) Spring c) Summer d) Fall.

**Figure 5:**
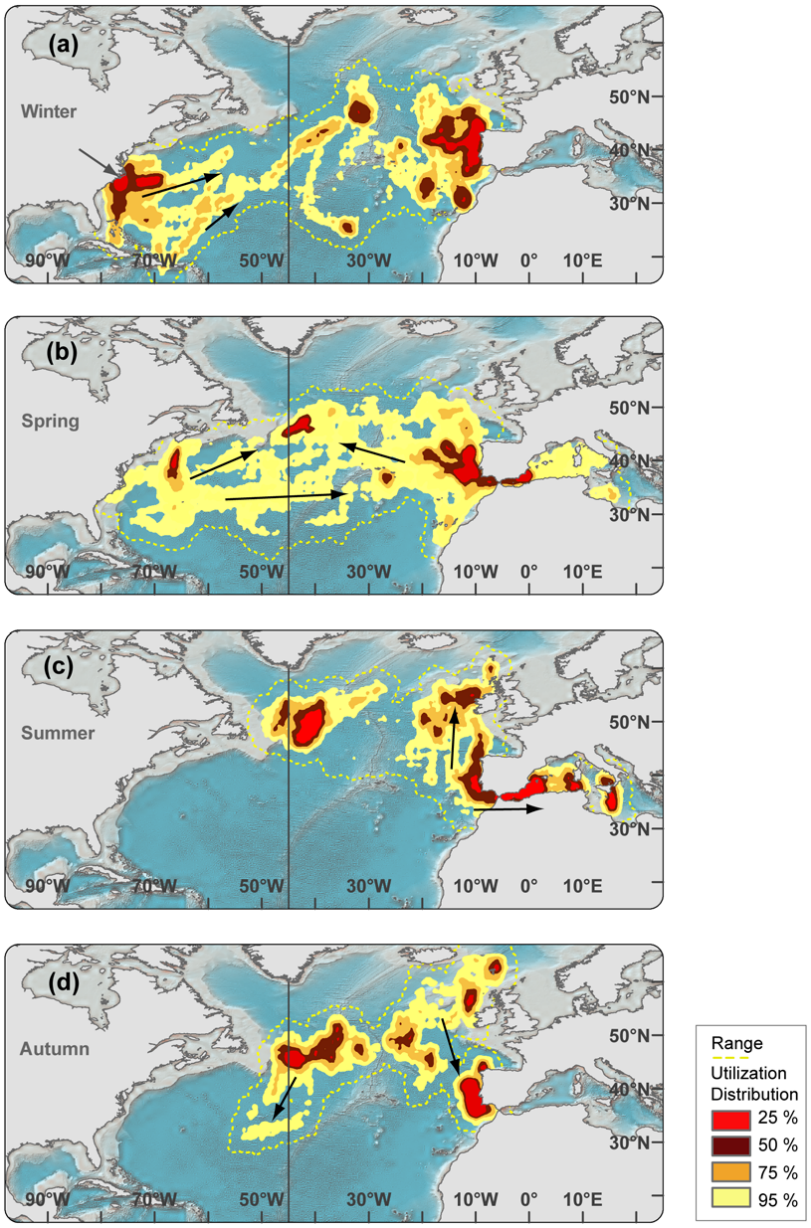
Seasonal utilization distributions of bluefin tuna in *trans-Atlantic* movement pattern (n = 21, 232±14 cm CFL). Black arrows in ocean depict general direction of movements during relevant season. (a) Winter. Grey arrow in North Carolina depicts approximate deployment location. (b) Spring (c) Summer (d) Fall.

In this study, distribution probabilities were calculated from the estimated geolocations using a tracking effort-weighted kernel density analysis to derive an index of tuna residence probability per unit area, to identify areas of multi-individual high utilization and to obtain real occupancy within these areas through extraction of the tracking data (Figure 3) in several steps: **1)** to provide for equally spaced tracks that could be pooled for analysis, gaps between consecutive dates were linearly interpolated to one position per day based on great circle distance. **2)** in order to factor the spatial error of the geolocations in the analysis, we randomly resampled each geolocation 100 times along the longitudinal (SD 0.78°) and latitudinal (SD 0.90°) error distribution (Gaussian) reported [20]. **3)** to retain the detail of the distribution patterns the kernel smoothing parameter h was selected by identifying the standard deviation from the minimum successive distance between resampled geolocations (mean±std, 0.3±0.5°). We opted to keep h constant, as opposed to an adaptive kernel, to be able to visually compare residence probabilities from different ocean regions. For visualization purposes the grid size was set at one-hundredth of the value of h i.e. 0.01 of a degree. **4)** the density surface derived from simple kernel analysis needed to be adjusted to reflect equal sampling effort within each grid cell [33], [34]. Due to the single deployment location in this study and the varying individual tracking durations, the number of tracked animals decreases randomly with distance from the deployment location, depicting a sampling bias towards the Northwest Atlantic (Figure 3c). In this region the grid for the daily re-sampled geolocation estimates showed very high densities around the tagging location in North Carolina (352–456 pos./km^2^) and over the Grand Banks (283–351 pos./km^2^, Figure 3b). We normalized the skewed density estimate of days tracked in each cell (Figure 3b) by dividing it by the number of individual bluefin tuna tracked within each cell (Figure 3c). The resulting index reflects a mean probability of tuna residency over the analyzed time domain. **5)** In order to identify areas of multi-individual utilization we reclassified the grid of the numbers of animals tracked per unit area before executing step 4. The area outlining 95% of animals tracked shows the distribution of at least three animals tracked (Figure 3c). The minimum number of animals permitted in the sampling effort grid was therefore reclassified to 5% of the dataset. In this way we down-weight cells frequented by less than 3 individuals and avoid biasing our identification of multi-individual high-use areas. **6)** The resulting multi-individual residence probability grid ultimately allowed the calculation of utilization distributions (UD) as a polygon coverage using least-squares cross validation [35], [36]. This provided probability contours that indicate the relative area utilized by the tracked fish over the time domain of the data analyzed. First we identified the high-use areas in the North Atlantic as the areas corresponding to the 25% utilization distributions of the entire tracking dataset (1996–2006, Figure 3d). These were used to query the tracking dataset and obtain true residence times within the high use areas as well as the natal origin of individuals. Secondly, we examined the seasonal utilization distributions of western resident and transatlantic fish (Figure. 4 and 5). Seasons were delimited by the respective solstices and equinoxes. Kernel density analysis for grid coverages and cell-based statistics were performed using ModelBuilder in ArcGIS 9 (ESRI).

It was previously determined that when using kernel estimators in an analysis of habitat utilization, the collection of more frequent locations within the same region may result in increased autocorrelation between points [37]. However, several authors [38]–[42] have argued that adequate sample size is more important than independence between points and it was therefore suggested that >50 positions would be adequate to avoid this problem [37]. Although the spread of locations can still be autocorrelated to some degree, the effects of spatial autocorrelation on the derived time spent per unit area, is likely to be reduced by correcting for tracking effort.

#### Oceanography of high-use areas

We examined whether the presence of bluefin tuna within the identified high-use areas coincided with specific physical (abiotic) and biological (biotic) settings that would define them and indicate their importance as foraging habitats. Sea Surface temperature was used as the abiotic parameter and we obtained an 8-day averaged SST product mapped at 4 km equal angle grids from the Pathfinder project data archive in the Physical Oceanography Distributed Active Archive Center (PODAAC, http://podaac.jpl.nasa.gov). Estimates of vertically integrated primary productivity (PP), which indicates the net biomass of primary producers present, were used as the biotic parameter. PP data was obtained as 8-day averages at a 0.1 degrees equal-angle grid served by the OceanWatch live access server of the NOAA Coastwatch and Environmental Research Division (http://las.pfeg.noaa.gov/oceanWatch/).

**Figure 6:**
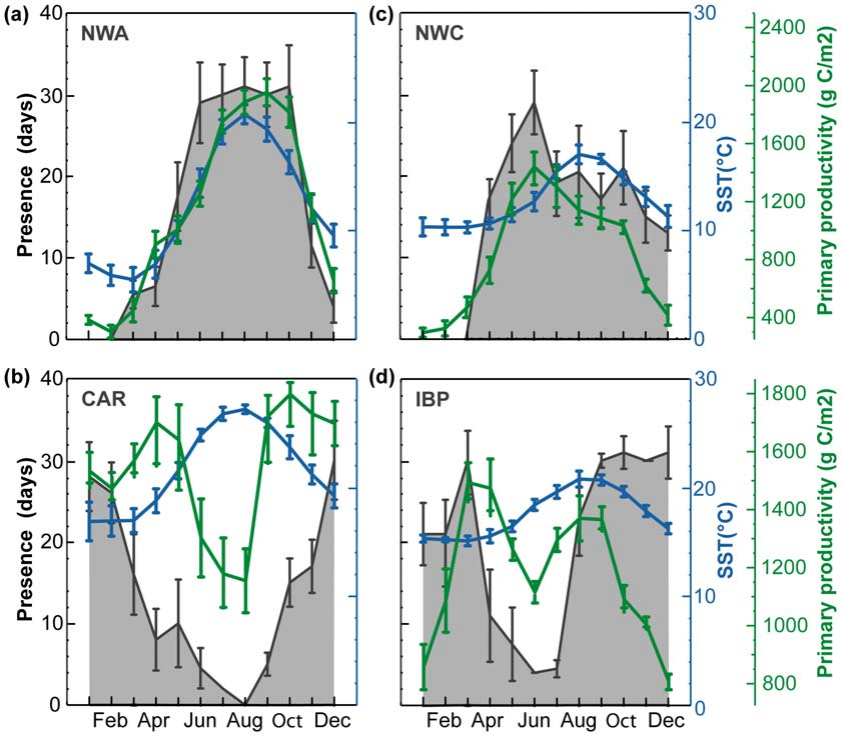
Mean (±SD) monthly number of days that bluefin tuna were present (1996–2005) within high use areas (grey shaded) in relation to mean (±SD) monthly level of primary productivity (green line) and sea surface temperature (blue line). (a) Nortwest Atlantic (n = 32) (b) Nortwestern Corner (n = 5) (c) Carolina (n = 52) (d) Iberian Peninsula (n = 4).

First, we calculated the mean number of days per month that the fish were present within each high-use area polygon of the 25% UD with the standard deviation measuring the difference between years (1996–2005, Figure 6). We then queried the remotely sensed sea surface temperature (SST) and derived primary productivity (PP) estimates present within each high-use area polygon corresponding to the tracking period (1996–2005). For each parameter we calculated the mean values per month with the standard deviation measuring the difference between years (Figure 6). The mean monthly presence of bluefin tuna was then analyzed in relation to the mean values of SST and PP using a least- square-fit regression [43].

#### Temperature and depth distributions

Time-series data from WC and Lotek tags allowed for three temperature and depth analysis: an overall, a high-use area specific and a water-mass specific. First, we calculated the minimum, maximum and mean of ambient temperature (Ta), body temperature (Tb) and depth data during the entire tracking time for each functional tag recovered (Table 2).

**Table 2:** Descriptive statistics for functional recovered archival tags with timeseries data (n = 44, 184–276 cm CFL).

Tag	Release Date	*Ta Days*	Ta (C°) Min	Ta (C°) Max	Ta (C°) Mean	Ta (C°) StdDev	*Tb Days*	Tb (C°) Min	Tb (C°) Max	Tb (C°) Mean	Tb (C°) StdDev	*Depth days*	Depth (m) Max	Depth (m) Mean	Depth (m) StdDev
97-016	3/7/1997	*59*	3.00	25.40	20.62	3.23	*95*	13.50	29.40	23.17	2.61	*95*	693	30.35	55.72
97-017	3/7/1997	*71*	4.60	24.50	17.49	4.83	*313*	15.20	30.20	24.05	2.63	*313*	786	28.20	49.34
97-019	3/7/1997	*92*	4.40	24.80	18.15	4.27	*344*	12.90	30.50	24.21	2.74	*344*	789	25.71	42.55
97-027	3/7/1997	*32*	6.00	24.70	19.56	2.51	*374*	12.40	31.70	24.08	2.85	*374*	787	29.84	57.67
97-028	3/7/1997	*69*	3.20	26.60	17.93	3.49	*389*	12.80	31.80	23.84	2.55	*389*	766	22.81	44.24
97-030	3/7/1997	*59*	3.20	25.40	16.59	3.49	*371*	12.50	31.20	23.86	2.95	*371*	770	34.31	53.14
97-037	3/7/1997	*11*	12.40	24.30	21.35	1.90	*261*	12.90	30.30	25.06	2.09	*261*	768	24.86	43.77
97-038	3/7/1997	*72*	3.00	25.20	19.56	3.98	*383*	13.30	32.00	24.97	2.48	*383*	789	42.27	58.35
97-048	3/7/1997	*50*	5.20	24.90	19.57	3.06	*280*	13.00	31.40	25.61	2.37	*280*	794	40.01	73.00
97-067	3/8/1997	*5*	15.80	25.90	21.19	0.89	*379*	13.20	32.10	24.50	2.90	*379*	790	29.03	38.23
97-011	3/16/1997	*19*	11.50	24.50	22.28	1.41	*139*	12.10	30.70	23.27	2.83	*139*	759	38.94	61.58
97-022	3/17/1997	*115*	3.00	25.57	18.24	4.23	*157*	15.30	31.30	23.56	3.09	*157*	789	35.85	60.97
97-089	3/17/1997	*42*	4.80	25.10	21.59	2.92	*315*	16.40	31.60	25.73	2.31	*315*	740	15.10	43.36
97-102	3/17/1997	*12*	13.40	24.70	21.99	1.71	*330*	14.80	32.20	24.80	2.21	*330*	785	30.30	27.84
97-103	3/17/1997	*20*	12.70	24.80	20.92	2.17	*363*	12.80	30.10	23.52	2.51	*363*	736	20.64	48.09
97-043	3/20/1997	*1*	13.10	23.60	20.32	7.50	*448*	14.20	31.50	24.02	2.78	*448*	656	36.24	55.64
97-112	3/21/1997	*42*	5.83	25.60	13.95	9.51	*68*	13.45	28.90	25.24	1.48	*68*	642	22.20	53.04
98-521	1/1/1999	*461*	3.00	25.60	16.55	4.72	*461*	11.00	31.80	24.34	2.35	*461*	989	73.98	136.34
98-492	1/6/1999	*468*	4.00	26.80	18.70	4.59	*625*	11.00	30.60	24.28	2.66	*625*	973	47.19	103.02
98-502	1/14/1999	*205*	3.00	25.80	18.12	4.46	*204*	15.00	33.50	23.60	2.83	*204*	899	37.33	68.56
98-510	1/14/1999	*55*	6.20	24.30	20.23	2.85	*55*	18.10	31.20	24.93	1.92	*55*	676	13.41	32.51
98-507	1/16/1999	*192*	3.00	28.20	16.72	5.50	*503*	11.10	32.30	23.14	3.11	*503*	820	66.55	97.21
98-518	1/16/1999	*136*	3.00	25.20	15.54	5.12	*451*	11.00	32.90	24.12	3.07	*451*	884	30.85	64.10
98-508	1/17/1999	*292*	3.00	26.70	17.68	4.41	*578*	13.60	30.60	24.07	2.66	*578*	994	32.61	62.34
98-512	1/17/1999	*237*	3.00	29.80	19.12	4.68	*583*	14.60	32.10	25.09	2.30	*583*	993	57.49	99.13
98-516	1/17/1999	*235*	4.40	25.80	17.58	4.06	*1598*	15.30	32.60	24.22	2.55	*1598*	996	43.05	73.51
98-485	1/21/1999	*480*	3.00	26.70	15.97	4.27	*478*	13.60	29.20	22.55	2.34	*478*	996	55.22	79.85
98-504	12/31/1999	*520*	3.40	25.60	16.93	4.59	*686*	11.00	26.30	20.65	2.59	*686*	882	51.34	87.88
14	2/1/2002	482	4.22	26.83	17.36	4.00	482	10.69	28.02	20.70	2.84	482	879	31.49	58.81
793	1/13/2003	78	10.12	25.24	21.22	1.94	398	14.12	29.94	23.79	2.65	398	855	30.47	51.66
1025	1/13/2003	327	0.04	26.11	16.84	4.14	327	13.86	31.48	24.57	2.79	73	234	32.40	3.19
781	1/14/2003	223	4.36	28.71	16.78	4.84	223	15.51	32.04	24.28	2.54	223	559	32.69	50.86
1013	1/16/2003	*108*	5.15	24.85	19.16	3.06	*108*	14.55	29.48	23.07	2.41	*108*	695	25.02	48.71
744	1/18/2003	385	1.48	28.27	17.47	4.77	385	14.82	29.61	23.23	2.50	385	721	23.07	41.48
1016	1/18/2003	396	0.04	29.29	17.50	4.47	396	12.95	30.23	23.48	2.81	396	995	46.09	89.24
1005	1/18/2003	526	3.43	26.71	18.31	4.50	526	11.29	32.19	23.97	2.84	526	1217	28.00	51.34
1000	1/21/2003	388	2.85	29.16	17.57	4.69	388	14.43	32.22	24.29	2.53	388	658	32.50	0.02
532	1/25/2003	510	1.53	29.81	17.80	5.32	720	9.66	30.81	24.41	2.24	720	925	48.14	90.98
566	1/25/2003	33	6.27	24.10	20.23	1.86	353	12.29	32.04	24.52	2.73	353	994	32.31	50.27
1021	1/26/2003	410	4.07	28.35	17.20	4.40	410	15.44	31.79	24.25	2.37	410	909	28.47	48.83
2217	1/9/2004	352	0.08	26.52	16.00	4.63	352	10.20	27.31	21.99	2.57	352	1107	37.52	75.65
2159	1/17/2004	29	15.46	24.28	21.27	1.39	525	15.08	33.11	23.35	2.73	416	119	18.81	14.81
2158	1/22/2004	433	3.08	26.21	16.57	4.35	594	12.14	31.28	22.87	3.02	594	775	26.75	55.34
219	1/12/2005	313	1.77	26.08	17.27	4.16	313	12.66	30.63	22.68	3.13	313	687	24.63	42.59
	**Total**	***8986***	**0.04**	**29.8**	**18.4**	**2.0**	***17636***	**9.7**	**33.5**	**23.9**	**1.1**	***17273***	**1217**	**34.5**	**12.8**

**Figure 7:**
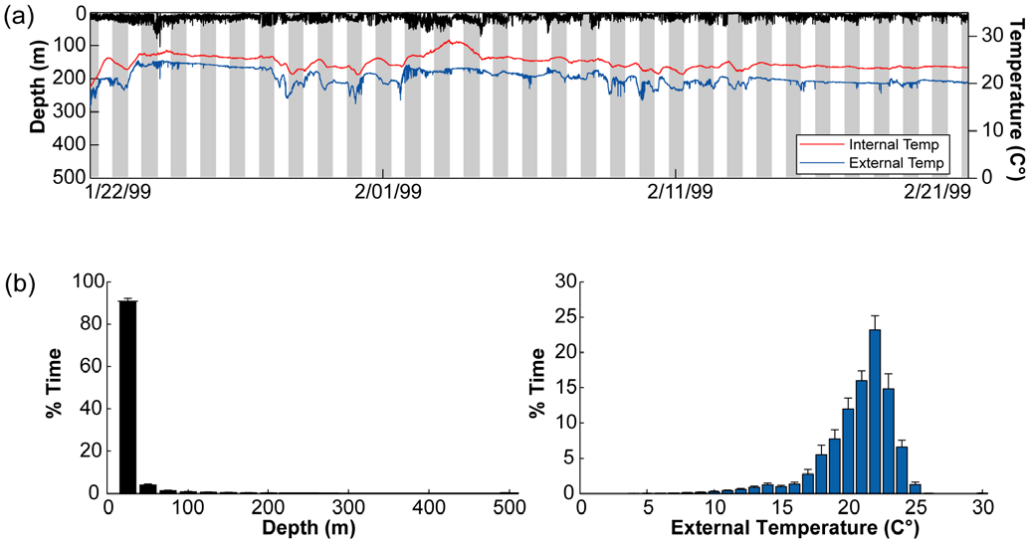
Typical diving behavior, external and internal temperature in the high use area of North Carolina. (a) One month of a typical diving behavior (black) profile displayed with external (blue) and internal temperature (red) (WC98-485). Grey shades indicate nighttimes obtained through light level data. (b) Overall depth (left,black histogram) and ambient temperature (right, blue histogram) preferences in the high use area of North Carolina (1997–2005; n = 50).

**Figure 8 pone-0006151-g008:**
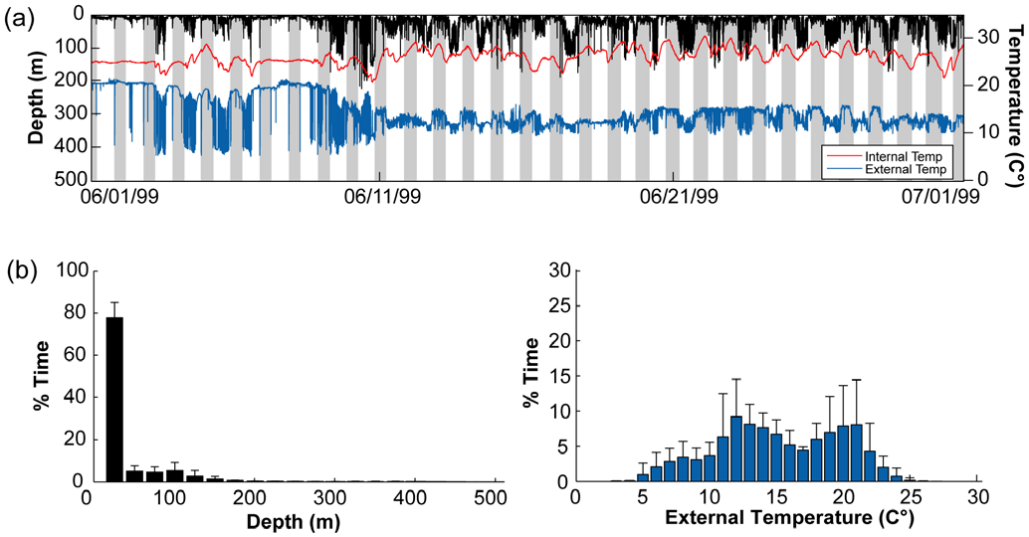
Typical diving behavior, external and internal temperature in the high use area of New England. (a) One month of a typical diving behavior (black) profile displayed with external (blue) and internal temperature (red) (WC98-521). Grey shaded region indicates nighttime obtained through light level data . (b) Overall yearly depth (left, black histogram) and ambient temperature (right, blue histogram) preferences in the high use area of the Northwest Atlantic (1997–2005, n = 26).

**Figure 9:**
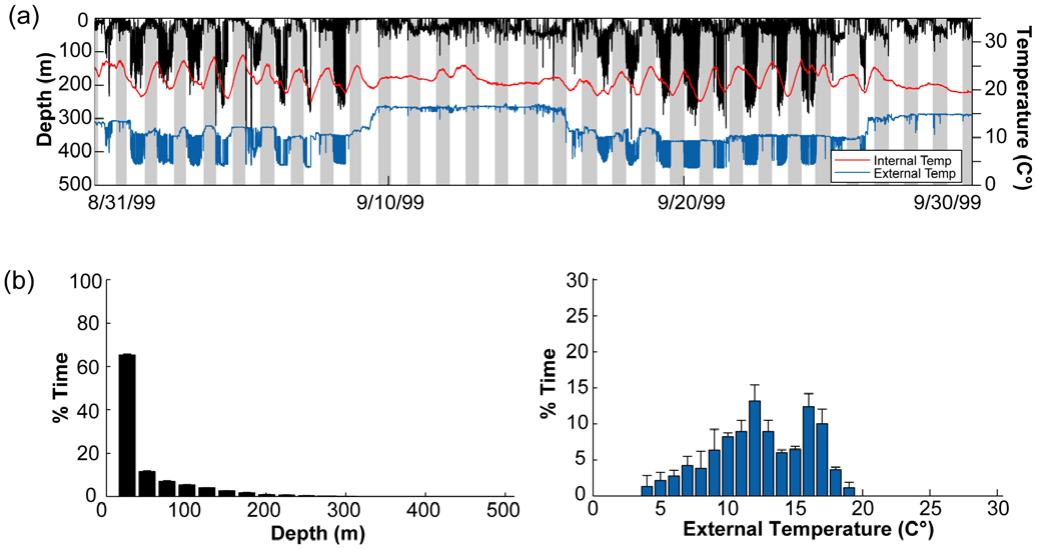
Typical diving behavior, external and internal temperature in the high use area of the North West Corner. (a) One month of a typical diving behavior (black) profile displayed with external (blue) and internal temperature (red) (WC98-485). Grey shades indicate nighttimes obtained through light data . (b) Overall yearly depth (left,black histogram) and ambient temperature (right,blue histogram) preferences in the aggregation area of the North West Corner (1999–2000; 2004, n = 4).

We then compared the depth, Ta and Tb data of the bluefin tunas between the identified high-use areas (25% UD) for the years available (Figure. 7, 8, 9; Table 3). Within each polygon for these high-use areas, the depth/temperature data for each fish present were parsed into bins and the averages reported in histograms. Standard deviations measured the difference between the means of individual fish. Time at depth and temperature distributions were compared using a Kolmogorov-Smirnov two sample test [30] to detect significant changes between years. Differences in temperatures experienced and diving behavior displayed were then analyzed between day and night.

**Table 3:** Summary of residency and composition of tracked ABFT as well as diving and temperature indexes within particular high use area and non-high use areas (1996–2005, mean±sd).

	CAR	NWA	NWC	IPB	Transit
**Years obtained**	1996–2005	1996–2005	1999–2000; 2004	1999–2005	1996–2005
**Residency per year (days)**	94±35	125±62	111±32	104±75	128±68
**Individuals tracked**	52	32	5	4	52
**Mean CFL (cm)**	209±30	214±24	236±7	247±10	232±14
**Western breedingstatus**	8	10	2	-	8
**Eastern breedingstatus**	23	9	2	4	23
**Neutral breedingstatus**	21	13	1	-	21
**Mean depth (m)**	16.6±6.5	34.1±7.5	44.0±3.6	n.a.	73.5±21.8
**Mean divefrequency**	15.3±5.8	22.9±4.7	26.0±1.6	n.a.	8.8±1.7
**Mean variance Ta (°C)**	8.8±3.2	21.0±5.2	11.2±3.1	n.a.	17.5±8.2
**Mean variance Tb (°C)**	5.1±1.5	8.4±3.0	5.9±1.9	n.a.	3.9±1.3

We compared the mean depth, dive frequency and variance in Ta and Tb between the high-use areas as well as to times of transit (Table 3) to establish whether the animals display a behavior that would be indicative of foraging. Increased diving activity has been previously described to be indicative of foraging behavior in bluefin tuna [4]. Here we defined diving frequency as the number of descents per day that were longer than 15 m in depth, regardless of the depth from which they started. Further, the digestion of food is associated with an increase in basal metabolism [44], [45] and in bluefin tuna the amount of increase in visceral warming (Tb) as measured through archival tags has been found to be proportional to the amount of food ingested [46], [47]. Here we employ the daily measured variance in Tb relative to the variance in Ta to obtain an indication of feeding activity. To isolate differences in obtained mean values of depth, dive frequency and variance in Ta and Tb between regions and during transit, we used a multi-comparison Analysis of Variance with a set of Bonferroni corrected t-tests [48] and reported when significant.

**Figure 10:**
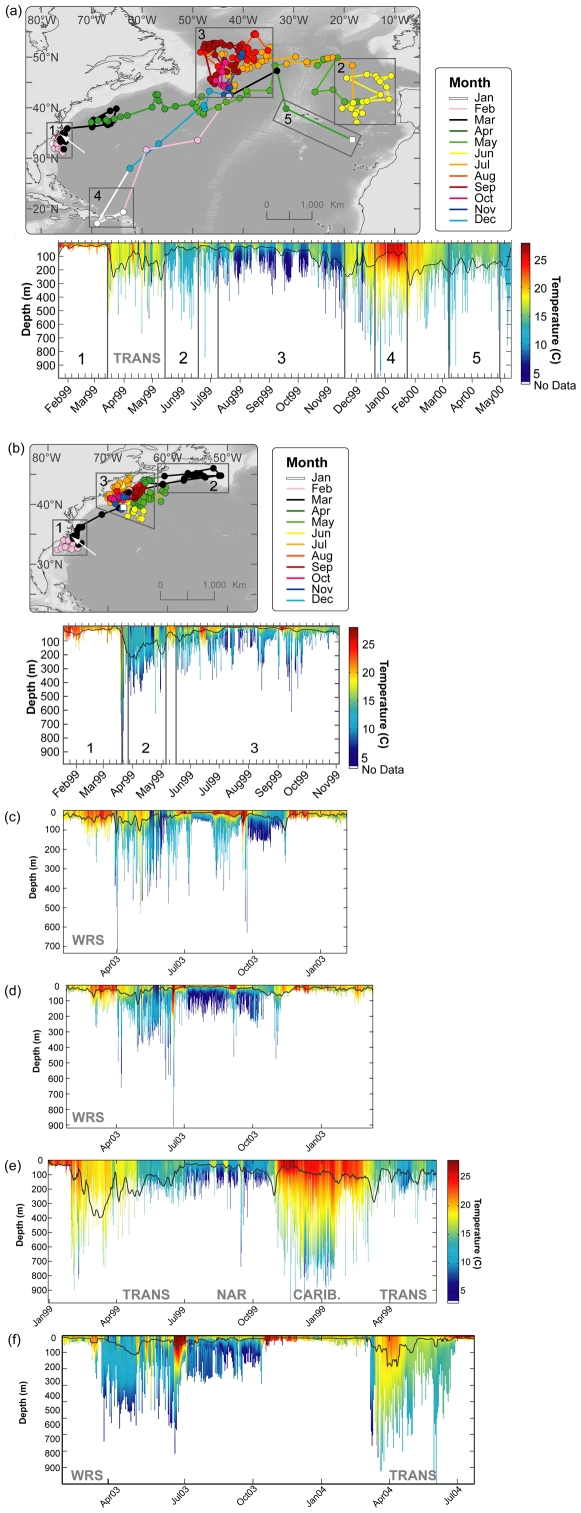
(a)–(b) Monthly geolocation estimates and track of individual Atlantic bluefin tuna with corresponding depth and temperature profiles indicating maximum diving behavior in relation to water temperature. Black boxes indicate geographic regions covered by profiles. White arrow indicates deployment location. Black line in depth/temperature profiles indicates estimated depth of thermocline. (a) Bluefin in North Atlantic resident migration (98–485). Section 1: North Carolina, 2: off offshore Iberian Peninsula, 3: in North Western Corner, 4: northern Caribbean, 5: East Atlantic passing through Azores. (b) Bluefin in western resident migration (98–508). Section 1: North Carolina, 2: south off New Foundland & Novia Scotia, 3: offshore New England, Gulf of Maine, Fundian Channel and then Georges Bank. (c)–(f) Examples of depth/temperature profiles of fish in various migration phases. (c) & (d) WRS (744 & 1021); (e) TRANS and North Atlantic residency (98–504); (f) WRS & TRANS (1016) with entry into the Mediterranean Sea in July.

**Figure 11:**
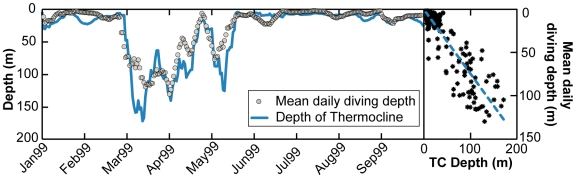
Example of relationship between mean daily diving depth (grey circles) and corresponding mean daily depth of estimated thermocline (blue line) for one fish (WC98-508; LSFR, R^2^ = 0.92; P<0.0001).

We examined diving behavior displayed by individuals along their tracks in relation to the temperature structure of the water column. For each 4 hr. time period a depth/temperature profile corresponding to the maximum diving depth was re-constructed with the average temperature experienced for each meter fitted using a locally weighted polynomial regression (loess fit; [49]). These depth/temperature profiles were stacked to show the differences in water-mass-specific diving depth between western resident and transatlantic migrant bluefin tuna (Figure 10). From these profiles we calculated the water-mass-specific vertical temperature gradients and the depth of the thermoclines. To estimate the depth of the thermocline (TC) for each profile we used a criterion of Δ1.0°C per 2 m and selected the depth at which this criterion first occurred [50, pers.comm.]. The daily mean, median and maximum diving depth were then analyzed in relation to the daily thermocline depth using a least-squares-fit Regression (LSFR, [43]) and reported when significant (Figure 11).

All statistical tests in this study were performed with the Statistics Toolbox in Matlab 7.0.1 (The Mathworks).

## Results

### Deployments and Recoveries

Bluefin tuna captured and released in North Carolina coastal waters in 1996–1999 had mean curved fork lengths (CFL) of 198.3±16 cm (mean±sd; n = 561). Fish tagged in 2002–2005 had a mean CFL of 203.2±19 cm, indicating they were significantly larger from the first cohort of tagged fish (Wilcoxon rank sum test, P<0.05; Figure 1). Overall, the size of the archival tagged bluefin tuna released between 1996–2005, based on measured CFL, ranged from 138 cm to 268 cm.

To date, 106 (19%) of the archival tagged bluefin tuna have been recaptured by commercial fishers from the 1996–2005 deployments (Supplementary material, Table 1) and recapture rates varied from 2–30% between years (Table 1). Recovery rate was higher for fish released in the period from 1996–1999 (26.3±5.5%) than from the 2002–2005 deployments (10±3.6%, Table2). Tagged fish spent on average 1,161±868 days at large before recapture (Supplementary material, Table 1).

The recapture positions of western tagged bluefin tuna (Figure 2) demonstrates that archival tagged fish were recaptured throughout the extent of the fishery across the North Atlantic and into both known spawning areas. Of the 106 recaptured archival tagged bluefin tuna, 42 (40%) were reported east of the 45 meridian stock boundary, and 29 (27%) were recaptured in the Mediterranean Sea.

Tag return and tag performance played a role in acquiring time series records from the archival tags. Although 106 recaptures were reported through the recapture of tuna and reporting of the associated floy tags, 81 archival tags were actually returned by fishers to scientists, and of these 62 tags recorded data. Physical failures included over-pressurization of early generation tags, water intrusion into the Teflon external light stalk, or tag body, broken thermistor wires and broken thermistor bulbs. Several tags had memory failures and one tag was cut up by a band saw at the Tokyo fish market. The pressure sensors on 42% of the Mk7 archival tags and 29% of the LTD2310 drifted and was corrected before analysis of the data, with the largest drift experienced being 18 m over 1.3 years

### Geolocation data

We obtained 561 GPS positions at deployment and 103 at recapture from fishers or scientists who recovered and reported the tags. In addition, 3 recapture positions had to be estimated based on the descriptive information on the location provided by the fishermen. For 57 individual bluefin tuna a total of 11,391 filtered longitude estimates spanning 1996–2006 were estimated, and for 52 bluefin tuna the combination of the daily light, depth and external temperature record allowed SST geolocation (Supplementary material, Table 1) and hence spatial analysis of movements. After linear interpolation of the filtered geolocation dataset (mean gaps 1.8±4.6 days) the mean track length was 368±139 days (n = 52). The longest track record spanned 1627 days (NMT603).

### Spatial distribution

#### Multi-individual high-use areas

The tracking effort corrected utilization distribution of bluefin tuna revealed four hot spot areas in the North Atlantic that were visited most frequently between 1996–2006 and in which western tracked bluefin tuna resided for extended periods (Figure.3d, 4b and 6; Table 3). Bluefin tuna were consistently tracked within the overall high-use area off the coast of North Carolina for 94±35 days per year, with fish aggregating in these waters from as early as mid October to as late as the middle of May depending upon the year (Figure.3, 4 and 6; Table 3). The months of highest residency in this region were December through March. Bluefin tuna were recorded in a second high-use area in the North Western Atlantic (Gulf of Maine, Georges Banks and south of Nova Scotia) for 164±62 days per year, with fish aggregating in this area from early March to late December (Figure.3, 4 and 6a; Table 3). The highest residency in this region occurred in June through October. In the central North Atlantic, a region of high-use was identified to the east of the Flemish Cap, known as the North Western Corner [49], for 167±33 days (Figure. 3d and 5; Table 3). Fish aggregated in this region as early as April and remaining through December, with peak occupancy in June (Figure. 5 and 6c; Table 3). In the Northeast Atlantic, a fourth high-use area was identified off the western coast of the Iberian Peninsula (Portugal and southwest Spain) where fish were consistently present 126±75 days per year (Figure. 3d and 4; Table 3). However, peak presence in this region occurred from September to December as well as in May (Figure.5 and 6d;). Bluefin tuna were absent from the overall high-use areas an average of 128±68 days per year with peak times of transit between these areas occurring during spring months (Table 3; Figure.4b and 5b). The size of fish (mean curved length) was significantly different between the high-use areas (multi-comparison ANOVA, P<0.05; Table. 3).

#### Movement patterns

Based on the annual longitudinal distribution of individuals (n = 57) we differentiated between tuna (n = 49) that displayed western residency (herafter called WRS) within the western North Atlantic (west of 45°W meridian, Figure 4) and individuals (n = 21) that moved trans-Atlantic (hereafter called TRANS, Figure 5) for the given year of tracking. Western residency as well as trans-Atlantic movements from west to east were consistently observed throughout the tracking years (Table 1) with minor variability in longitude distribution between years. Bluefin tuna in western resident phase had a relatively small coefficient of variation in longitudinal distribution (CV* = 5.4%) between years while the trans-Atlantic migration phase had higher variation (CV* = 20.2%).

#### Seasonal movement patterns connect high-use areas

In winter, western resident bluefin (WRS) aggregated in the high-use area off the North Carolina waters as well as south of Nova Scotia (Figure 4a). Individual tuna were also present ranging from the Grand Banks in the north to offshore waters of the Bahamas, Cuba and Puerto Rico in the south. The range (100% UD) of WRS bluefin was greatest in spring, extending westward into the Gulf of Mexico and eastward almost to the 45°W Meridian. In summer, the range retracted and a high-use area emerged over Georges Bank and the southern Gulf of Maine (Figure 4c), which remained there throughout autumn. During fall, fish migrated down the coast to aggregate in North Carolina but individual fish were also present in the Sargasso Sea and the northern Caribbean (Figure 4d).

The range (100% UD) of bluefin tuna undergoing trans-Atlantic migrations (TRANS) spanned the North Atlantic, from the North Carolina high-use area to the Mediterranean Sea. For bluefin tuna that had moved trans-Atlantic the previous year, the winter high-use area was off the Atlantic coast of the Iberian Peninsula from 45°N to 35°S (Figure 5a). Notably, departure time from the American Continental Shelf was correlated with latitude, starting in January from the Sargasso Sea and lasting to May from Nova Scotia. This coincides with the seasonal, latitudinal productivity regime of the North Atlantic [51] and might explain the variability in departure time (Figure. 5a and b). In summer the full range of all TRANS fish had moved from the Western to the Central and Eastern North Atlantic (Figure 5c). Individuals in the Central North Atlantic (n = 5) formed a large high-use area in the North Western Corner centered at 43°N–60°N (Figure 5b). In autumn, no western tagged bluefin tuna remained in the Mediterranean Sea, but individuals had moved back into the Atlantic aggregating off the Iberian Peninsula (Figure 4d). Two (NMT779, WC98-485) individuals were seen to migrate from the North Western Corner to the northern Caribbean where they remained for three weeks during the winter months before returning again to the North West Corner in spring (Figure. 4a and 9a).

### Abiotic and biotic factors in high-use areas

The average number of days per month that tracked bluefin tuna were present within each high-use area (Table 3) was related with the mean monthly patterns of SST and primary productivity between1996–2005 (Figure 6). Monthly SST's were significantly positively correlated with the presence of bluefin tuna in the high-use area of the North Western Atlantic (LSFR, P<0.01, R^2^ = 0.94) and negatively correlated in the North Carolina waters (P<0.01, R^2^ = −0.83; Figure 6). In the two northern high-use areas, the North Western Atlantic and the North Western Corner, the monthly level of primary productivity was highly correlated with the presence of bluefin tuna (P<0.01, R^2^ = 0.94; P<0.01, R^2^ = 0.77 respectively; Figure 6a, c). In the lower latitude high-use areas this relationship was weaker (CAR, P<0.01, R^2^ = 0.53) or not significant (IPB, P<0.24, R^2^ = 0.34).

### Ambient temperature and depth preferences

#### Overall temperature and depth preferences

The ambient water temperatures experienced by tagged bluefin tuna had a range of 0.04°–31.0°C and a mean that varied between 18.2°±2.0°C obtained from tags that recorded a complete time series (n = 44; 8,986 days; 202±13 cm CFL; Table 2) and 16±5°C obtained through binned data (n = 8, 8,748 days; 207±15 cm CFL). For the entire temperature dataset, the bluefin spent 87% of occupancy in waters ranging from 10° to 23°C with peak times at 13°–20°C (60%). The three Lotek tags that recorded the extreme minimum temperatures of 0.04–0.08°C (LTD1025, 1016, 2217) were factory inspected, recalibrated and showed full functionality of their sensors. At the time these low temperatures were recorded two of these fish were at the entrance of the Gulf of Saint Lawrence where the mean water temperature at 100 m is 1.23±1.00°C (Hydrographic database, Bedford Institute of Oceanography). While temperatures <1°C represent rare encounters, ambient temperatures around 2–4°C were commonly recorded during deep dives in waters off Nova Scotia and west of the Flemish Cap.

The internal body temperatures for bluefin reporting timeseries data showed a mean of 23.9°±1.1°C (n = 44; 17,636 days; Table 2) and 24°±1.6°C for tags reporting binned data (n = 8; 8748 days; 207±15 cm CFL).

Overall, the mean diving depths of bluefin tuna was 34.5±12.8 m (Table 2), with most of their time spent between the surface and 50 meters (79±8%; binned NMT data) and a exponential decrease in time spent at greater depths. Maximum depth in excess of 1,200 m was recorded by one fish (LTD1005, Table 2). Depth preferences of the 44 fish reporting time series data differed significantly between day and night (Kolmogorov-Smirnov test, P<0.05); fish spent more time in surface waters (<50 m) during the night than during the day.

#### High-use area specific temperature and depth preferences

The diving behaviors and water temperatures encountered by the archival tagged bluefin tuna were site specific and differed between the four overall high-use areas (Figs. 7–9a; Table 3). However, within the high-use areas the mean diving depth was significantly shallower and the dive frequency and the variance in internal temperature significantly higher than compared to times in transit outside the high-use areas (multi-comparison ANOVA, P<0.01; Table 3).

In the high-use area off North Carolina, diving behavior was limited by bathymetry, although deeper dives up to 550 m occurred when the fish moved on occasion offshore beyond the continental shelf (Figure 7; Figure 10, section1). Fish in this region spent ≥95% of their time within the upper 50 m and significantly more time near the surface (<10 m) during the day than during the night (Kolmogorov-Smirnov test, P<0.05). Depth and ambient temperature distributions in this region did not differ significantly among years (Kolmogorov-Smirnov test, P<0.05). Peak time was spent in waters of 20°–23°C (71%) with a range of 7 to 27.8°C (Figure 7b). There was no difference between day and night temperature preferences for any of the tagging years.

In the Northwest Atlantic (Gulf of Maine, Georges Banks and south of Nova Scotia), the largest of the observed high-use areas (Figure 3d), diving preferences and thermal data were highly variable (Figure 8) and most likely influenced by season and location. In spring (Apr-May) the fish were either located offshore associated with the northern wall of the Gulf Stream or in colder inshore waters over the Continental shelf south off Nova Scotia (Figs. 4b and 10b). In June-July many bluefin tuna started to move inshore over Georges Bank, showing a much shallower diving distribution (91.2±4% of time at 0–50 m), with a further inshore movement into the Gulf of Maine as the season progressed (Figure. 4c and 10b–d). In Oct-Nov, these bluefin moved out of the Gulf of Maine and occupied waters offshore from Georges Bank to Nova Scotia. Depth and temperature distributions differed significantly among years (Kolmogorov-Smirnov test, P<0.05) which was likely a result of different seasonal tracking times between the years. For all fish aggregating in this region, there was no significant difference of diving depth between day and night.

In the North Western Corner east of the Flemish Cap (Figure 3d), bluefin tuna displayed a very distinctive diving behavior in relation to water masses encountered. In the cold water of the North Wall (3–13°C, 42.5% total occupancy), repetitive dives to 50–300 m were made during the day (61.2±7.1% of time) with significantly more time (Kolmogorov-Smirnov test, P<0.05) spent between 0–50 m after sunset (95.1±2.8% of time; Figure 9). Here the depth of the thermocline was between 50 (summer)–200 m (fall). In the comparatively warm North Atlantic Current (15–19°C, 29% of time; Figure 9) bluefin showed an irregular diving behavior that was most often limited to the upper 50 m (98.1±1.4% of time) with no difference between night and day. Overall, a significantly deeper depth distribution was displayed on the cold side (<15°C) of the front and shallower diving on the warm side (Kolmogorov-Smirnov test, P<0.05; Figure 10a, section 3).

The overall high-use area off the western coast of the Iberian Peninsula (Figure 3d) was occupied by bluefin tuna tagged with NMT tags which were set to return binned data after 3 months of sampling to avoid memory shortage on long-term missions. Therefore, no dive and temperature analysis could be performed for this region.

#### Diving behavior in relation to thermal structure of water column

Depth/temperature profiles were used to reconstruct the ocean water column profiles (8,986 daily profiles; n = 44) to obtain estimation of the depth of the thermocline and water masses encountered by bluefin tuna (Figure 10). While the maximum diving depths were limited by bathymetry during on-shelf phases, they were highly variable in relation to thermal structure of the water column in both on- and off-shelf phases (Figure 10). However, the time spent at depth was influenced by the degree of stratification of the water column. In the Gulf of Maine, for example, the preference for surface waters (91.2±4% of time at 0–50 m) was associated with the highly stratified water column (Δ°C 0.2±0.03°C/m) characterized by a very shallow thermocline (TC 12±4.2 m; n = 25; Figure. 10b, c and d end Jul.-Aug.). In contrast, tuna that moved trans-Atlantic entered the weakly stratified (Δ°C 0.06±0.01°C/m; TC 38±22 m) water mass of the Northeast Atlantic Boundary Current (14±1.5°C) where they spent less time (64.2±7%, n = 4) above the thermocline (Figure 10a, section 2). In summary, we found the mean diving depth of bluefin tuna to be significantly correlated (LSFR, P<0.01; R^2^ = 0.72; n = 44) with the depth of the thermocline throughout the North Atlantic (Figure. 11). In waters with a shallow thermocline, fish remained significantly shallower in mean depth, while in waters with a deeper thermocline they occupied deeper mean depths.

## Discussion

The deployment and recovery of electronic archival tags from 1996 to 2006 on western tagged Atlantic bluefin from ages 7.1 to 14.2 years provides a long-term observation series. In this study we employed this dataset to examine the seasonal movements, aggregations and diving behaviors to better understand their migration ecology and oceanic habitat utilization.

### Bluefin tuna horizontal and vertical focal areas

Distribution behavior was characterized by seasonal aggregations and rapid movement phases. Throughout the North Atlantic, high residence times (167±33 days) were identified in four spatially confined regions on a seasonal scale. Within these areas, the bluefin tuna (219±20 cm) display unique diving behavior with significantly shallower diving depths and higher dive frequencies as compared to times in transit (Table 3; Figure 3d, 7–9). Moreover, the visceral temperatures (Tb) of bluefin tuna within these areas showed a significantly higher variance that occurred independently of the variation in external temperature (Table 3). The magnitude of variances in Tb within the high-use areas suggest an increase in visceral warming events likely caused by higher feeding activity. High-use areas likely represent critical foraging habitats where tuna can access enough prey to satisfy their energetic needs and remain within their preferred temperatures.

The location and timing of the high-use areas in the North Atlantic revealed by electronic tags coincides with favorable biophysical settings and the timing of high prey availability in each area of aggregation. Seasonality of prey availability in these foraging habitats necessitates migration between them. Starting at the deployment location, the presence of bluefin tuna over the Continental Shelf in North Carolina region (Figure 5a) was documented both historically through catch in the offshore waters of North and South Carolina region by Japanese longliners [54], [55], and more recently through acoustic and pop-up satellite tagging technologies [5], [7], [10], [21], [56]. Boustany [24] showed that the presence of bluefin tuna coincides with a large number of prey species, namely Atlantic menhaden (*Brevoortia tyrannus*), spot (*Leiostomus xanthurus*) and Atlantic croaker (*Micropogonias undulates*), that spawn in this region at the bottom during the night between November and March in waters of 18–24°C [57]. Multi-year records obtained through archival tags in this study show ambient temperature preferences of bluefin tuna in the region to be consistently between 20–23°C and the tuna to have a significantly deeper depth distribution during the night (Figure 6); further, the records show that bluefin tuna have fidelity to the region, return by October and reach peak residence in the months of December and January (Table 3; Figure 6a). The repeatability of these patterns year to year [5,18,this study] and the strong seasonal patterns of movements, suggest that there is a predictable food supply attracting the tunas to this region. Multi year stomach content analyses of bluefin tuna caught in this region revealed Atlantic menhaden to be the most common (96%) prey item [58]. The attraction of bluefin tuna to these menhaden spawning aggregations might also explain the de-coupled link to primary productivity and the weaker relationship observed in this region (Figure 6b). Specifically, the local spring bloom coincides with a breakdown of the cross-shelf thermal gradient which has been attributed to aggregate high densities of prey items [59]. Here, the warming of SST's beyond 21°C (Figure 6b) was consistently linked to the spring departure of bluefin tuna [23] which might explain the strong correlation between monthly SST's and residence days in this region.

By the end of March the first bluefin tuna in western resident phase arrived in the New England and Scotian Shelf waters. These fish stayed in the warmer offshore waters during spring months and it is not until the early summer months that most of the tuna moved into the Gulf of Maine area (Figure 4c). These movements follow closely the known migrations of pelagic forage fish (eg.herring species, mackerel species) and squid specis from offshore waters across Georges Bank into the Gulf of Maine [60]. Here dramatic increases in primary productivity and sea surface temperatures during the summer and fall month (Figure 6a) foster favorable habitats that attract and support a high abundance of prey species. Specifically, the presence of bluefin tuna in this region has been linked to the summer & fall aggregations of mackerel (*Scomber scombrus*), herring (*Clupea harengus*), squid species [16], [61]–[64] and sandlances (*Ammodytes americanus*; [15], [65]. Hence, the residence of bluefin tuna in this region was highly correlated with increase in primary productivity and sea surface temperatures (Figure 6a). While the diving behavior in the Gulf of Maine was dependent on the location, we found that it was generally characterized by shallower dives associated with well stratified waters (Figure. 10b, c and d end Jul.-Aug.). The strong thermal stratification may provide the physical means to aggregate primary food sources for prey (Figure 10b, c, d, summer months) and allow easier detection, access and successful encounters for bluefin tuna forage in this region.

Some bluefin tuna of larger body size demonstrated a trans-Atlantic migration pattern into the central North Atlantic that was distinct from the direct west to east movement (Figure 10a; Table 1). Starting in spring they migrated to the region in North Western Corner (NWC) where they resided for up to 8 month (Figure. 5a, b, c and 6c). The long residence times of bluefin tuna in this area are supported by the consistently high-catch-per-unit-effort results of the Japanese longlining fleet in this region [66]. Considering these extensive migration movements (>6000 km), the energetic revenue of foraging in this region must be very high. It is well established that the region north of 45°N is the site of very productive spring blooms which are associated with mesoscale eddies and meanders that concentrate the primary productivity to support very large secondary and tertiary trophic biomasses [67]–[70]. Here monthly primary productivity patterns were also directly related to the time spent by tracked bluefin tuna in this study (Figure 6c). Depth and temperature records (Figure 9a; 10a, section 3) indicate that bluefin in the NWC display a unique foraging behavior in relation to the North Wall of the North Atlantic current that can be identified by diving behaviors, ambient temperature and thermoregulatory biology observed through the archival tag records. Significantly deeper dives occurred on the cold side of fronts during the day, potentially to access mesopelagic fish species. The diving behavior displayed in the region appears to be a foraging behavior because body temperature (Tb) shows numerous events of sharp thermal decline at a fast rate, that potentially indicate peritoneal cooling upon ingestion of cold prey [18], [71]. These bluefin repeatedly moved back and forth across frontal features on a weekly basis (Figure 10a, section 3). While residing in the warm North Atlantic current they showed reduced diving activity with no diel behavior and few changes in Tb.

Western tagged bluefin tuna of very large size (247±10 cm CFL, n = 16) exhibited a direct trans-Atlantic movement (TRANS) during spring month (Figure. 5b, 10f, section TRANS). This shift in residence from the Northwest Atlantic into the eastern Atlantic was age dependent and only individuals larger than 200 cm (CFL, ∼8.1 years of age) at the time of trans-Atlantic movement showed this behavior [10]. While residing in the East Atlantic these fish displayed aggregations off the Atlantic coast of the Iberian Peninsula (IBP), the Azores, Ireland and remote offshore locations over the Mid-Atlantic Ridge (Figure 5a–d). However, all tagged bluefin tuna in eastern resident phase spent considerable time (126±75 days) off the IBP, where they showed the highest presence from fall to winter and in spring (Table 3, Figure 6d). Upwelling and primary productivity peak in IBP waters during spring and fall month [72]–[75] attract spawning aggregations of sardines [76] and high abundances of mackerel species [77] and blue whiting [78]. Individuals residing off the IBP were subsequently tracked to known spawning grounds in the Mediterranean Sea (n = 12).We hypothesize that the highly productive waters off the IBP act as an important foraging region for large, mature bluefin tuna on their way to and from spawning grounds in the Mediterranean Sea [87].

The focus in this paper has been to highlight the foraging aggregations and provide an overall view of the distinctive behavior and biology in these regions. Here, the depth/temperature analyses were determined by the movement patterns of individuals as opposed to previous observations of diving behavior in relation to the continental shelf [5], [7]. There were also clear distinctions in the diving behaviors in relation to the thermal structure of the water column throughout the North Atlantic (Figure 10). Overall, the mean diving depth of bluefin tuna in the North Atlantic was significantly correlated with the depth of the thermocline (Figure 11). The thermal stratification at the depth of the thermocline provides physical means to vertically aggregate food for prey species [79], [80]. By focusing diving depths around the thermocline Atlantic bluefin tuna potentially maximize the probability of encountering prey. Such a strategy was further reflected in the diel behavior away from the continental shelves where there was a strong affinity for deeper waters during the day, when the deep scattering layer is at depth. Overall, the observed vertical and horizontal movement behaviors suggest an optimization towards maximizing forage encounter. The ability to adapt to the variability of prey abundance in time, space and vertical dimension is essential to bluefin tuna as a highly migratory pelagic predator.

### Interpretation of dispersal patterns

Characteristics of the geolocation dataset, ontogenetic changes of movement patterns and the size range of the fish tracked require a cautious interpretation of the spatial distribution patterns revealed. The overall patterns that emerge however are striking as they reveal key hot spots for adolescent and mature bluefin tuna in the North Atlantic.

The assessment of the inter-annual variability in distributions of tracked bluefin tuna was hindered by a significant variation in the number of geolocation estimates obtained per year (Table 1). Nonetheless, both western residency and the trans-Atlantic movements were consistently observed in each year using longitude records and recapture positions alone (Table 1). The inter-annual variability in longitude distributions of the trans-Atlantic migration patterns was found to be higher than that of the western resident migration pattern. This was mainly due to 1) variation in departure times between years; 2) incomplete trans-Atlantic tracks due to recapture or tag failure and 3) a higher proportion of transatlantic movements during the 2002–2005 period in comparison to the 1997–2001 period.

The latter requires consideration of the measured size and natal origin of the tracked bluefin tuna. The data on length indicate that the electronic tagging of younger fish in the earlier years of the program (1997–2001) may have resulted in a higher proportion of western residency recorded by immature fish of eastern origin, which subsequently moved to or were recaptured at known spawning grounds in the Mediterranean Sea; these fish (n = 12) were significantly smaller than western fish (n = 10; Wilcoxon rank sum test, P<0.05, mean CFL 210 and 222 cm respectively). In contrast, during 2002–2005 tagged fish were of significantly larger size and displayed a higher trans-Atlantic movement rate which resulted in increased recapture rates in the Mediterranean Sea. Supported by the observations of size related dispersal patterns by [10], [12] and [87] this provides further evidence for the ontogenetic change in movement patterns that is also evident in western fish [10], [19].

Further, ontogenetic movement patterns need to be considered when comparing results from tracking studies to distributions obtained through CPUE data. The size range of fish tagged in this study and the relatively small proportion of animals tracked might explain the lack of high-use areas identified in the known fishing grounds such as Canada and the Bay of Biscay. For example, in recent years distribution patterns of a larger cohort (248±12 cm) of PSAT tagged bluefin tuna off North Carolina showed a range expansion and residency in the more northern waters of Canada (Block, et al., unpublished data) that is also manifest through a recent increase in CPUE's in this fishery [81]. However, the mean size of tagged bluefin tuna in this study was well below that caught in the Canadian fishery (>250 FL cm, [80]) and well above that for the Bay of Biscay (54–105 FL cm, [82]), hence reduced the likelihood of identifying these fishery regions as high-use areas.

### Management implications

The high concentrations of bluefin tuna in predictable locations indicate that Atlantic bluefin tuna are vulnerable to concentrated fishing efforts in the regions of foraging aggregations. This has important implication for national and international management of the fishery. Bluefin tuna electronically tracked spent an average of 246±51 days per year in the spatially confined high-use areas identified in this study (Table 3). This aggregation behavior indicates that the CPUE of a particular fishery (e.g. Purse Seining) most likely will serve poorly as an index for population abundance [83]–[85]. Future biomass estimations of adults or juveniles should consider spatiotemporal variation in abundance when drawing on population indices.

Our studies have shown clear evidence of mixing between eastern and western populations in foraging aggregation zones [5], [10], [86], which are well supported by recent findings through otolith based stable isotope analysis [87]. Identifying the degree of mixing within an aggregated region will increasingly be of high importance. Genetic testing of DNA samples collected during release will in the future be useful for assessing population status [86], [88] and potentially confirming the ratio of western fish to eastern fish in all regions of high utilization. Ideally, natal origin and age dependent spatial components should be incorporated in the stock assessments to facilitate more realistic biomass estimates. Both electronic tracking data and DNA analyses can aid in the development of future models that incorporate this information.

For trans-Atlantic bluefin tuna that returned to the Mediterranean Sea there was a high chance of capture. Only 10% of all fish moving into the Mediterranean Sea were not caught and could be tracked beyond the spawning season exiting the Mediterranean Sea. These fish (n = 4) remained in the eastern Atlantic close to their natal spawning ground, potentially to maximize their reproductive output. Because of their annual spawning behavior [10] and the associated high reproductive value, the protection of these large fish is probably of particular importance to the conservation management of the eastern stock.

The documented high-use areas may represent a network of critical foraging habitats that are essential collectively to the persistence of bluefin tuna populations in the North Atlantic. Further, the apparent dependence of their seasonal movements on the productivity and high abundance of prey species in a given high-use area potentially makes the presence of bluefin tuna sensitive to changes in regional ecosystem productivity. Sibert et al. [12] hypothesized recently that bluefin tuna migrations and inter-annual shifts noted with satellite tagging datasets may in part be linked to shifts in oceanographic conditions. In this study, with numerous multi-year tracks, there appear to be repeatable patterns of clear aggregation areas that potentially are changing with environmental conditions. The apparent stability of these regions to be seasonally productive may provide predictable foraging habitats for bluefin tuna. Identifying the underlying processes that make the seasonal ecosystem productivity of oceanic regions predictive for pelagic animals, versus areas that are highly ephemeral will aid in our understanding of identifying vital bluefin tuna habitat.

## Supporting Information

Table S1 Release and recapture information of archival tagged Atlantic bluefin tuna.
(0.05 MB XLS)
